# An omics-based machine learning approach to predict diabetes progression: a RHAPSODY study

**DOI:** 10.1007/s00125-024-06105-8

**Published:** 2024-02-19

**Authors:** Roderick C. Slieker, Magnus Münch, Louise A. Donnelly, Gerard A. Bouland, Iulian Dragan, Dmitry Kuznetsov, Petra J. M. Elders, Guy A. Rutter, Mark Ibberson, Ewan R. Pearson, Leen M. ’t Hart, Mark A. van de Wiel, Joline W. J. Beulens

**Affiliations:** 1 Department of Epidemiology and Data Science, Amsterdam UMC, Vrije Universiteit, Amsterdam, the Netherlands; 2 Amsterdam Public Health, Amsterdam, the Netherlands; 3 Amsterdam Cardiovascular Sciences, Amsterdam, the Netherlands; 4 Department of Cell and Chemical Biology, Leiden University Medical Center, Leiden, the Netherlands; 5 Population Health & Genomics, School of Medicine, University of Dundee, Dundee, UK; 6 Delft Bioinformatics Lab, Delft University of Technology, Delft, the Netherlands; 7 Vital-IT Group, SIB Swiss Institute of Bioinformatics, Lausanne, Switzerland; 8 Department of General Practice, Amsterdam UMC, Vrije Universiteit, Amsterdam, the Netherlands; 9 CRCHUM, Faculty of Medicine, Université de Montréal, Montréal, QC Canada; 10 Department of Metabolism, Digestion and Reproduction, Faculty of Medicine, Imperial College London, London, UK; 11 Lee Kong Chian School of Medicine, Nanyang Technological University, Singapore, Republic of Singapore; 12 Department of Biomedical Data Sciences, Section of Molecular Epidemiology, Leiden University Medical Center, Leiden, the Netherlands; 13 Julius Center for Health Sciences and Primary Care, University Medical Center Utrecht, Utrecht, the Netherlands

**Keywords:** Machine learning, Prediction model, Progression, Type 2 diabetes

## Abstract

**Aims/hypothesis:**

People with type 2 diabetes are heterogeneous in their disease trajectory, with some progressing more quickly to insulin initiation than others. Although classical biomarkers such as age, HbA_1c_ and diabetes duration are associated with glycaemic progression, it is unclear how well such variables predict insulin initiation or requirement and whether newly identified markers have added predictive value.

**Methods:**

In two prospective cohort studies as part of IMI-RHAPSODY, we investigated whether clinical variables and three types of molecular markers (metabolites, lipids, proteins) can predict time to insulin requirement using different machine learning approaches (lasso, ridge, GRridge, random forest). Clinical variables included age, sex, HbA_1c_, HDL-cholesterol and C-peptide. Models were run with unpenalised clinical variables (i.e. always included in the model without weights) or penalised clinical variables, or without clinical variables. Model development was performed in one cohort and the model was applied in a second cohort. Model performance was evaluated using Harrel’s C statistic.

**Results:**

Of the 585 individuals from the Hoorn Diabetes Care System (DCS) cohort, 69 required insulin during follow-up (1.0–11.4 years); of the 571 individuals in the Genetics of Diabetes Audit and Research in Tayside Scotland (GoDARTS) cohort, 175 required insulin during follow-up (0.3–11.8 years). Overall, the clinical variables and proteins were selected in the different models most often, followed by the metabolites. The most frequently selected clinical variables were HbA_1c_ (18 of the 36 models, 50%), age (15 models, 41.2%) and C-peptide (15 models, 41.2%). Base models (age, sex, BMI, HbA_1c_) including only clinical variables performed moderately in both the DCS discovery cohort (C statistic 0.71 [95% CI 0.64, 0.79]) and the GoDARTS replication cohort (C 0.71 [95% CI 0.69, 0.75]). A more extensive model including HDL-cholesterol and C-peptide performed better in both cohorts (DCS, C 0.74 [95% CI 0.67, 0.81]; GoDARTS, C 0.73 [95% CI 0.69, 0.77]). Two proteins, lactadherin and proto-oncogene tyrosine-protein kinase receptor, were most consistently selected and slightly improved model performance.

**Conclusions/interpretation:**

Using machine learning approaches, we show that insulin requirement risk can be modestly well predicted by predominantly clinical variables. Inclusion of molecular markers improves the prognostic performance beyond that of clinical variables by up to 5%. Such prognostic models could be useful for identifying people with diabetes at high risk of progressing quickly to treatment intensification.

**Data availability:**

Summary statistics of lipidomic, proteomic and metabolomic data are available from a Shiny dashboard at https://rhapdata-app.vital-it.ch.

**Graphical Abstract:**

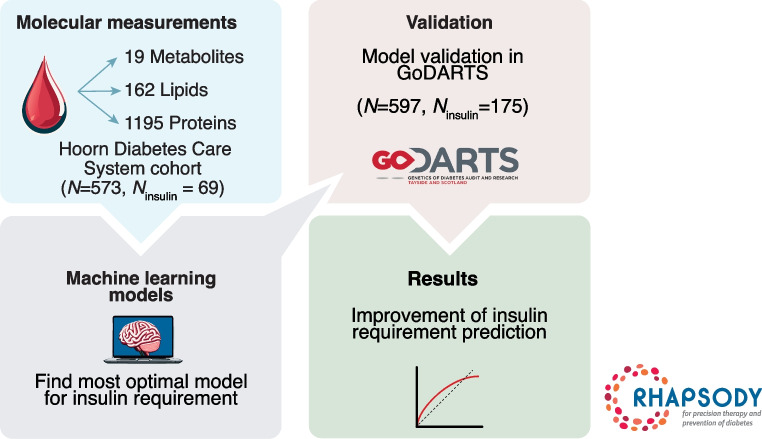

**Supplementary Information:**

The online version contains peer-reviewed but unedited supplementary available at 10.1007/s00125-024-06105-8.


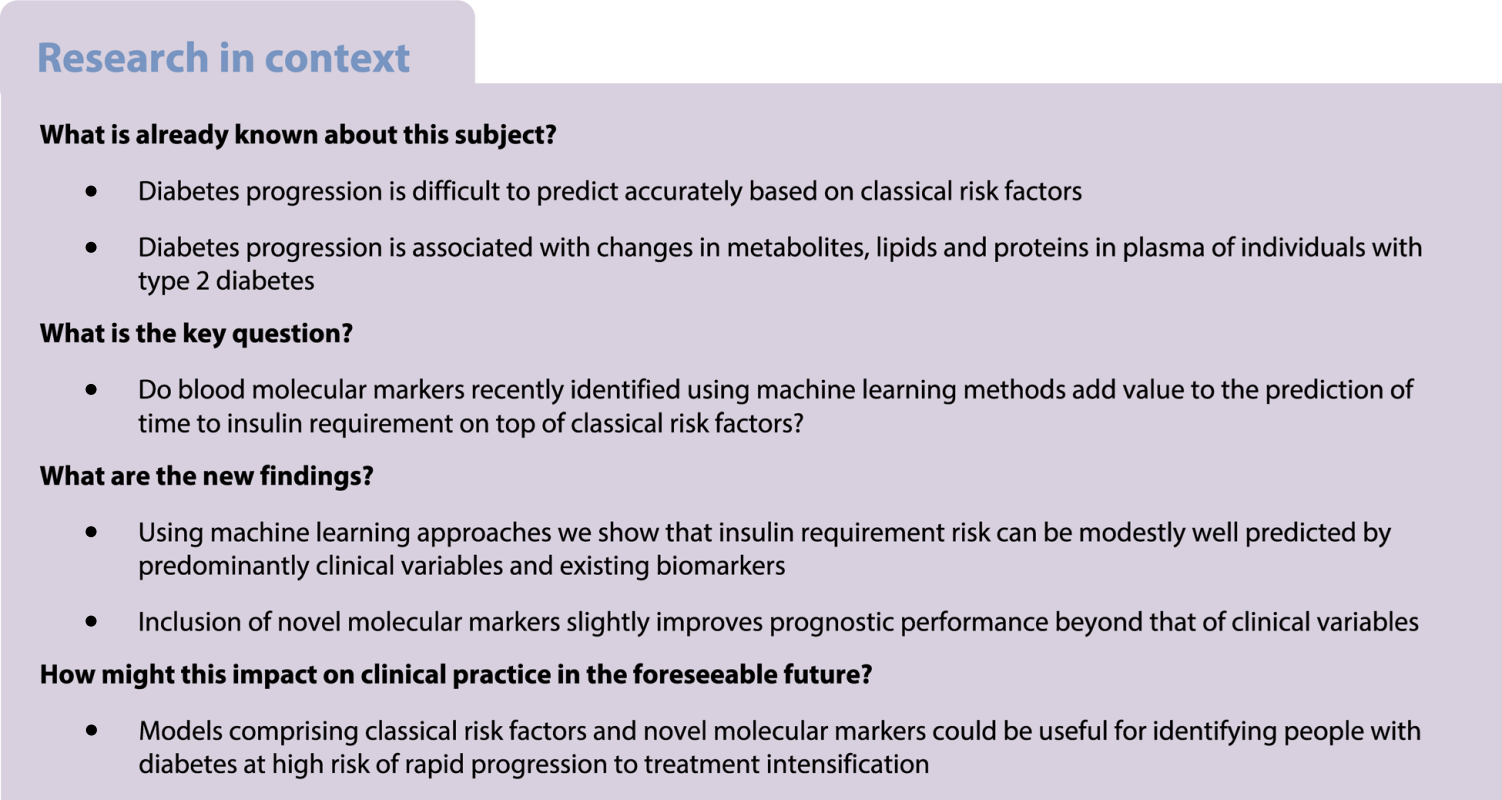


## Introduction

People with type 2 diabetes are heterogeneous in their disease trajectory. While some progress quickly to insulin, others show good glycaemic control for decades using only oral glucose-lowering drugs. Ideally, one would identify those at risk of progressing quickly to initiation of treatment intensification to prevent glycaemic progression and development of complications. Therefore, many studies have investigated predictors of glycaemic progression, treatment intensification, initiation of insulin or requirement for insulin. These studies showed that baseline HbA_1c_, young age and weight gain were independently associated with these outcomes in different populations [1–5]. Other important predictors in some of these studies included diabetes duration, complications or levels of blood lipids, such as HDL-cholesterol or triacylglycerol [1, 3–5]. Although these studies reported independent associations between these determinants and different outcomes of glycaemic progression, they did not investigate how well such risk factors predicted risk of progression in terms of identification of high-risk individuals (discrimination) or quantifying the risk of progression (calibration).

As genetics play a key role in the development of diabetes, and pharmacogenetic studies have identified certain variants associated with poor response to certain glucose-lowering drugs, several studies have investigated the genetic predictors of glycaemic progression. These studies showed that polygenic risk scores including known diabetes variants were associated with a young age at diagnosis or insulin initiation [3] or with rapid progression to insulin therapy [1]. Such findings may provide insight into the pathophysiological pathways underlying glycaemic progression.

In a previous study, we identified biomarkers for time to insulin requirement using a univariate approach based on proteomics, metabolomics and lipidomics [6]. This study showed that biomarkers from different molecular classes, including triacylglycerol, multiple proteins and small charged metabolites, were associated with time to insulin requirement. However, these univariate analyses do not identify the most informative combination of biomarkers with which to predict time to insulin requirement. Moreover, they do not account for the added prognostic value beyond that of known predictors of progression, such as age, HbA_1c_ or BMI. In recent years, an increasing number of studies have adopted machine learning approaches to identify optimal sets of predictors for specific outcomes, and take into account additional information such as the type of biomarkers included in the selection model [7]. In the current study, we build on our previous work and develop prediction models for time to insulin initiation using previously identified clinical variables and three types of molecular measures (small charged metabolites, lipids and proteins). For this, we explored different machine learning approaches to identify the most optimal model in the Hoorn Diabetes Care System (DCS) cohort and validated the models in the Genetics of Diabetes Audit and Research in Tayside Scotland (GoDARTS) cohort.

## Methods

### IMI-RHAPSODY

RHAPSODY (https://imi-rhapsody.eu/) is a European collaboration based around EU Innovative Medicines Initiative-2. As part of RHAPSODY, three different biomarker classes were measured, that is, charged small molecules (metabolites), lipids and proteins, in three large cohorts (DCS, GoDARTS and All New Diabetics In Scania [ANDIS]).

### Cohorts

Data from two cohorts, DCS (the Netherlands) and GoDARTS (Scotland), were used in the current study. Criteria for inclusion in this study have been described previously [6]. Briefly, individuals were included when the age of diagnosis was ≥35 years, when clinical data were available within 2 years after diagnosis and they were GAD negative, and when genome-wide association study (GWAS) data were present. Descriptions of the DCS and GoDARTS can be found in their respective cohort profiles [8, 9]. In short, the DCS is an open prospective cohort study from the north-west part of the Netherlands that started in 1998 and includes over 14,000 individuals with type 2 diabetes. The Ethical Review Committee of the Vrije Universiteit Medical Center, Amsterdam, approved the study and informed consent was obtained from all participants. Laboratory variables and molecular measures (metabolomics, lipidomics, proteomics) in the DCS were obtained from a sample collected in the fasted state. GoDARTS includes people with type 2 diabetes from the Tayside region of Scotland (*N*=391,274; January 1996) who were added to the Diabetes Audit and Research in Tayside Scotland (DARTS) register [9]. Data were collected from medical registries, including data on prescribing, biochemistry and clinical data. Laboratory variables and molecular measures in GoDARTS were obtained from a sample collected in the non-fasted state. The Tayside Medical Ethics Committee approved the GoDARTS study and informed consent was obtained from all participants. Cohorts were representative of the larger cohorts from which they were sampled. The self-reported race and ethnicity of individuals was collected and people were mainly of European descent. Sex was self-reported and both men and women were included in the study.

### Endpoint

The primary endpoint in the current study was time to insulin requirement. Time to insulin requirement was defined as prescribed insulin use or the requirement of insulin, with the latter defined as HbA_1c_ >69 mmol/mol (8.5%) when on two or more non-insulin diabetes therapies. In the DCS, medication data were obtained from dispensing labels on medication that participants brought to their annual monitoring visit. In GoDARTS, prescribing data were obtained from registries.

### Clinical covariates

Clinical variables included age, sex, HbA_1c_, HDL-cholesterol and C-peptide. In the DCS, HbA_1c_ was measured annually using the turbidimetric inhibition immunoassay for haemolysed whole EDTA blood (Cobas c501; Roche Diagnostics, Mannheim, Germany). HDL-cholesterol (mmol/l) was determined enzymatically (Cobas c501; Roche Diagnostics). In GoDARTS, these measurements were collected from the registry. C-peptide was measured in both the DCS and GoDARTS on a DiaSorin Liaison (DiaSorin, Saluggia, Italy). In GoDARTS, all laboratory measurements were measured in a non-fasted state.

### Biomarkers

Three molecular data types were included in the model (i.e. small charged metabolites, lipids and proteins) and are described in more detail elsewhere [6]. Nineteen small charged metabolites were measured using ultra-high-performance LC–tandem MS (UHPLC-MS/MS). Lipids (162 lipids) were measured on the Lipotype lipidomics platform (Dresden, Germany) and proteins (1195 proteins) on the SomaLogic SomaScan platform (Boulder, USA).

### Statistical analysis

Baseline characteristics are provided for the DCS and GoDARTS cohorts separately. Continuous variables were summarised using median (IQR). Categorical variables were summarised as percentages. Eight types of model were initially investigated for DCS data, with time to insulin requirement as the outcome, to find the best prediction model. We also used the models to rank variables based on the number of times they were selected, to give some indication of their relevance for the endpoint studied.

The following models were included:

- least absolute shrinkage and selection operator (lasso)
- (regular) ridge
- empirical Bayes group-regularised ridge (GRridge)
- empirical Bayes group-regularised ridge with selection (GRridgesel)
- cross-validated group-regularised ridge (multiridge)
- empirical Bayes group-regularised lasso (GRlasso)
- (regular) random forest
- co-data regularised random forest (CoRF)

Lasso and ridge (both fitted with the glmnet package, https://glmnet.stanford.edu/articles/glmnet.html) are regularisation methods that shrink the regression coefficients towards zero, where lasso may set them to zero (automatic variable selection). Here, lasso and ridge were combined with Cox regression. They both require one tuning parameter that was estimated by cross-validation (i.e. predictive partial likelihood maximisation). GRridge [7] and multiridge [10] are similar to regular ridge but estimate one tuning parameter per feature group (clinical variables, metabolites, lipids, proteins) by empirical Bayes and cross-validation, respectively. As such, they take the different types of markers and predictor variables into account and this may improve prediction. GRridgesel is an extension of GRridge and implements a post hoc step that selects features through the addition of a lasso penalty term to the ridge model [7]. Alternatively, GRlasso uses the penalty weights estimated by GRridge in the estimation of a lasso model [11]. The random forest is a non-linear (less interpretable) machine learner that fits many (1000 in the current study) decision trees to a random selection of predictors and predicts from new data by averaging over these decision trees. CoRF [12] is similar to the regular random forest but estimates predictor-specific sampling probabilities by considering extra information on the predictors (i.e. marker type). For both random forest types we changed the node splitting rule from logrank to logrankscore to yield better-calibrated survival curves. We included these learners because they all have different strengths. Random forest can deal with non-linear effects and interactions. Lasso caters for sparse settings in which only a few features matter (plus it performs feature selection). Ridge accounts for dense settings (many small effects) and allows for accounting for different feature groups (multiridge, GRridge).

All models were fitted in three different versions, differing in their use of clinical variables; the first version excluded penalisation of the clinical variables (model 1; M1); the second version penalised the clinical variables just as the other markers (model 2; M2); and the third version excluded the clinical variables from the model altogether (model 3; M3). Exclusion of clinical variables penalisation in the random forest was achieved by using large-feature sampling weights for the decision trees. Finally, we also fitted a model with only classical risk factors. Models were performed on the population for which all data types were available (i.e. the set with metabolomics, lipidomics and proteomics). The features comprised clinical covariates, including age, sex, creatinine, HbA_1c_, HDL-cholesterol, LDL-cholesterol, C-peptide, 15 metabolites, 135 lipids and 1195 peptides. The features were standardised before analysis to ensure that shrinkage of the coefficients was on the same scale. The eight methods that were applied included lasso, ridge, GRridge, GRridgesel, multiridge, GRlasso, random forest and CoRF. In total, 14 fits were applied: (1–3) lasso with the automatic variable selection and with 10 or 50 variables; (4) ridge; (5) GRridge with automatic variable selection; (6) GRridge combined with lasso; (7, 8) GRridge with 10 and 50 variables; (9–11) GRlasso with automatic selection and with 10 and 50 variables; (12) multiridge; (13) random forest; and (14) CoRF. The R package glmnet was used for lasso and ridge, the GRridge package for GRridge, the multiridge package for multiridge, the CoRF package for CoRF. For ridge models, post hoc feature selection was performed as described in van de Wiel et al [7]. The date of accession for the packages to fit the models was 5 November 2020.

Models were cross-validated ten times in the DCS to obtain performance estimates. GRridge, GRlasso and lasso models were subsequently replicated in GoDARTS. Discrimination of models in the discovery and validation cohort was based on Harrel’s C statistic, which is a measure for prediction performance ranging from zero to one. A C statistic of 0.5 indicated that the model did not perform better than random chance, whereas values above 0.7 indicated a good model and above 0.8 a strong model.

## Results

Characteristics of the DCS and GoDARTS cohorts are shown in Table 1. Both cohorts comprised people with type 2 diabetes who were followed longitudinally. Most individuals were male (DCS, 56.7%; GoDARTS, 59.1%), over 60 years of age (DCS, median 63.2 [IQR 56.2–70.3]; GoDARTS, median 61.9 [54.1–70.2]) and mainly treated with glucose-lowering drugs (DCS, 76.3%; GoDARTS, 55.7%). The endpoint of the study, time to insulin requirement, was reached in 69 individuals in the DCS out of the 585 individuals (follow-up 1.0–11.4 years) with all three measurements (metabolites, lipids and proteomics) and in 175 out of the 571 individuals in GoDARTS (follow-up 0.3–11.8 years).

**Table 1:** Characteristics of the DCS and GoDARTS cohorts

Variable	DCS	GoDARTS
*N*	573	597
Male sex (%)	56.7	59.1
Age, years	63.2 (56.2–70.3)	61.9 (54.1–70.2)
BMI, kg/m^2^	30.2 (26.7–33.1)	32.3 (27.7–35.8)
HbA_1c_, mmol/mol	46.9 (42.0–49.7)	55.5 (48.0–61.0)
HbA_1c_, %	6.4 (6.0–6.7)	7.2 (6.5–7.7)
C-peptide, nmol/l	1.2 (0.8–1.4)	2.2 (1.4–2.7)
HDL-cholesterol, mmol/l	1.2 (1.0–1.4)	1.3 (1.1–1.5)
LDL-cholesterol, mmol/l	2.6 (2.0–3.3)	2.2 (1.6–2.7)
Triacylglycerol, mmol/l	1.8 (1.1–2.2)	2.3 (1.4–2.8)
Diabetes duration at sampling (years)	1.6 (1.1–2.1)	0.9 (0.3–1.4)
Glucose-lowering medication use (%)	76.3	55.7

Data are presented as median (IQR) unless indicated otherwise

**Fig. 1:**
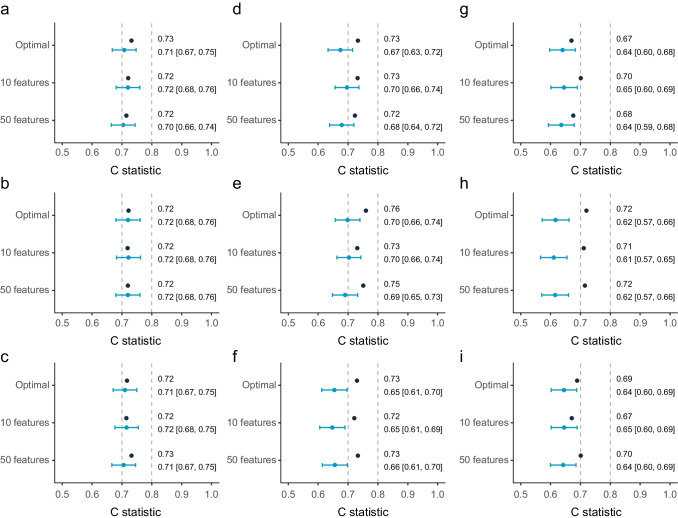
Comparison of model performance in the DCS and GoDARTS cohorts. Unpenalised (**a**-**c**) or penalised (**d**-**f**) clinical variables were included in the models or were excluded (**g**-**i**) from the models. Three methods were compared: GRlasso (**a**, **d**, **g**); GRridge (**b**, **e**, **h**); and lasso (**c**, **f**, **i**). Results for the DCS are shown in dark blue and results for GoDARTS in light blue. Dashed lines on the *x*-axis indicate good (C statistic >0.7) or strong (C statistic >0.8) performance. 95% CIs are shown in parentheses

Fourteen models were fitted on the DCS with time to insulin requirement as the outcome (Table 2) with (M1, M2) and without clinical variables (M3) and with the clinical variables unpenalised (M1) and penalised (M2). Overall, the clinical variables and proteins were selected in the different models most often (nine models), followed by the small charged metabolites. Lipids were selected in only three models (electronic supplementary material [ESM] Fig. 1). Among the most frequently selected clinical variables were HbA_1c_ (18 models; ESM Fig. 2, ESM Table 1), age (15 models) and C-peptide (15 models). Of the metabolites, isoleucine (26 models) and asymmetric dimethylarginine (SDMA/ADMA; 23 models) were selected most frequently. Isoleucine is a branched-chain amino acid and SDMA/ADMA are nitric oxide synthase inhibitors. From the proteins, proto-oncogene tyrosine-protein kinase receptor (RET; 27 models; ESM Table 1), C-C motif chemokine 14 (CCL14/HCC-1; 24 models) and IL-18 receptor 1 (IL18Ra; 23 models) were most often selected.

**Table 2:** Cross-validated C-index estimates in the DCS cohort

Model^a^	Unpenalised	Penalised	Exclusion
Lasso+sel (cv)	0.72	0.73	0.69
Lasso+sel (10)	0.72	0.72	0.67
Lasso+sel (50)	0.73	0.73	0.70
ridge	0.72	0.69	0.68
GRridge	0.73	0.72	0.69
GRridge+sel (lasso)	0.72	0.76	0.72
GRridge+sel (10)	0.72	0.73	0.71
GRridge+sel (50)	0.72	0.75	0.72
GRlasso+sel (cv)	0.73	0.73	0.67
GRlasso+sel (10)	0.72	0.73	0.70
GRlasso+sel (50)	0.72	0.72	0.68
MR	0.74	0.73	0.71
RF	0.71	0.67	0.67
CoRF	0.73	0.63	0.59

^a^Numbers in parentheses indicate the number of features selected in the model, that is, optimal number (cv) of features, 10 features or 50 features

cv, cross-validation; MR, multiridge; RF, random forest; sel, selection

### Discrimination and validation of models

Base models (age, sex, BMI, HbA_1c_) including only clinical variables performed modestly in both the DCS discovery cohort (C statistic 0.71 [95% CI 0.64, 0.79]) and the GoDARTS replication cohort (C 0.71 [95% CI 0.69, 0.75]). A more extensive model that also included HDL-cholesterol and C-peptide performed better in both cohorts (DCS, C 0.74 [95% CI 0.67, 0.81]; GoDARTS, C 0.73 [95% CI 0.69, 0.77]). The discrimination of investigated models with molecular measures is shown in Table 2. Models that included clinical variables generally performed better than models that excluded clinical variables. The best discrimination was observed for GRridge with penalised clinical variables and feature selection with lasso with a C statistic of 0.76 in the DCS (Fig. 1). This model contained four clinical variables, four metabolites and 15 proteins (ESM Table 1). The clinical variables HbA_1c_ (weight 0.08), age (weight −0.03), C-peptide (weight 0.03) and HDL-cholesterol (weight −0.02) were included. For the metabolites, isoleucine (weight 0.04), SDMA/ADMA (weight 0.03), indoxyl-sulfate (IndS, weight 0.03) and phenylalanine (weight 0.02) were included. For the 15 proteins, the strongest contributors were RET (weight 0.03), IL18Ra (weight 0.03) and apolipoprotein M (ApoM, weight −0.02). Insulin itself was also included (weight 0.02).

The poorest discrimination was achieved for the CoRF model with ‘penalised’ (weighted) clinical variables (C=0.63) or exclusion of clinical variables (C=0.59, no clinical variables). Validation of the models in GoDARTS showed that the most consistent results were observed for the unpenalised models, while the penalised models generally showed higher C statistic values in DCS than in GoDARTS. The performance of the unpenalised sparse models (~10 variables) was similar, with a C statistic of 0.72 in both DCS and GoDARTS and seven overlapping variables (Fig. 1, ESM Fig. 3). Among these were the expected clinical variables age, sex, C-peptide, HDL-cholesterol, HbA_1c_ and two proteins, lactadherin (milk fat globule-EGF factor 8 protein [MFGM]) and RET. Of note, while RET was selected in most models, lactadherin was selected in 18 models (ESM Fig. 2). In univariate meta-analyses, however, neither lactadherin nor RET was significantly associated with insulin requirement. Higher RET levels were associated with higher risk for insulin requirement (HR 1.29 [95% CI 0.92, 1.81], *p*=0.14), although both cohorts in the meta-analysis showed heterogeneity (*I*^2^=0.84) [6]. Higher levels of lactadherin were associated with a lower risk for insulin requirement (HR 0.90 (95% CI 0.60, 1.33], *p*=0.59) [6]. The models that excluded the clinical variables generally did not perform as well as the models with clinical variables included, with C statistics around 0.69. There were seven variables that were selected in nine models out of the 12 models with clinical variables excluded. These include isoleucine, RET, IL18Ra, hepatocyte growth factor receptor (MET), melanoma-derived growth regulatory protein (MIA), cystatin M and ApoM (ESM Fig. 4).

### Biomarkers are correlated with clinical variables

**Fig. 2:**
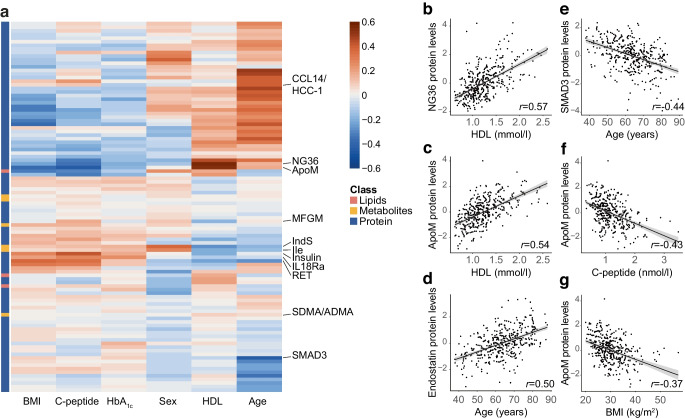
Correlation between clinical markers and biomarkers. (**a**) Heatmap of the correlation between clinical markers and biomarkers. (**b**–**g**) Scatter plots showing NG36 protein levels vs HDL-cholesterol levels (**b**); ApoM protein levels vs HDL-cholesterol levels (**c**); endostatin protein levels vs age (**d**); Mothers against decapentaplegic homolog 3 (SMAD3) protein levels vs age (**e**); ApoM protein levels vs C-peptide levels (**f**); and ApoM protein levels vs BMI (**g**). Protein levels are *z*-scaled. HDL, HDL-cholesterol; Ile, isoleucine; NG36, histone-lysine N-methyltransferase

The added value of biomarkers to the included models was modest. In part, this was the result of correlations between clinical markers and studied biomarkers. Indeed, correlations were observed for several of the biomarkers (Fig. 2a), particularly for HDL-cholesterol, BMI, C-peptide and age. HDL-cholesterol levels were positively correlated with histone-lysine N-methyltransferase (NG36 [also known as EHMT2], *r*=0.57; Fig. 2b) and ApoM levels (*r*=0.54; Fig. 2a,c) and negatively with diacylglycerols and triacylglycerols (ESM Fig. 5). Protein levels that altered with age included endostatin (*r*=0.50, Fig. 2d) and mothers against decapentaplegic homolog 3 (SMAD3, *r*=−0.44; Fig. 2e). ApoM levels also negatively correlated with C-peptide (*r*=−0.43) and BMI (*r*=−0.37; Fig. 2f,g).

## Discussion

In the current study, we used machine learning approaches to identify the most optimal model for time to insulin requirement from a compendium of three types of molecular measurements: small charged metabolites, lipids and proteins. We showed that models generally gave a modest prediction of time to insulin requirement, with C statistics ranging from 0.70 to 0.75. The most frequently selected variables were HbA_1c_, age, C-peptide, isoleucine, SDMA/ADMA, RET, CCL14 and IL18Ra. As expected, models that included the clinical variables generally performed better than models that completely omitted clinical variables. Inclusion of molecular markers improved predictive performance by up to 5%. Replication of the models was reasonable in GoDARTS, particularly when the clinical variables entered the model unpenalised.

This study shows that a model consisting of clinical variables and several selected biomarkers performs reasonably well to predict time to insulin requirement. Previous studies have not investigated the predictive performance of selected predictors for glycaemic progression to insulin initiation or requirement. We can thus not compare our findings with those of previous studies. Nevertheless, in our study, age, HbA_1c_ and C-peptide were the most frequently selected clinical variables, similar to findings of previous studies [1, 3–5]. In addition, model performance improved when including HDL-cholesterol and C-peptide, also in line with previous studies [1, 3–5]. Replication in GoDARTS showed reasonable prediction when clinical variables were included unpenalised, but the prediction was diminished in the other models. Nevertheless, the results were comparable overall with those of the DCS. The models that showed the best discrimination in the validation in GoDARTS were based largely on the same variables, with seven variables that overlapped across models (i.e. age, sex, C-peptide, HDL-cholesterol, HbA_1c_ and two proteins lactadherin and RET). For RET, higher levels were associated with higher insulin requirement risk in the univariate analysis. In previous studies, RET has been associated with cardiometabolic endpoints. Higher levels of RET were associated with higher risk of the metabolic syndrome and a Mendelian randomisation in the same study suggested a causal relationship [13]. Lactadherin is transcribed from the *MFGE8* locus and has been shown to be present in microvescicles excreted by different cell types including dendritic cells and adipocytes [14, 15]. In the latter, exposure to high levels of glucose increased the levels of lactadherin [15]. This is in line with the current study wherein higher lactadherin levels were associated with higher risk of insulin requirement.

Compared with our previous discovery study, there was limited overlap with the identified markers in the current study. For the metabolites, isoleucine and SDMA/ADMA were selected most often but these were not necessarily the top metabolites in our previous study [6]. We did, however, show in our previous study [6] that plasma isoleucine levels are significantly associated with prevalent and incident diabetes. For the most frequently selected proteins, only IL18Ra overlapped with the top identified proteins in our previous study [6]. The small overlap may be due to collinearity with the clinical variables, correlation among the omics variables or differences between the included cohorts.

Regarding the three distinct types of molecules, the metabolites and proteins were more often selected into the models than the lipids. We observed that ApoM and NG36 levels correlated with lipid levels. ApoM was selected far more often than lipids (19 models) and was selected more often than NG36 (two models). ApoM is a lipoprotein and more than 95% of the ApoM is bound to HDL-cholesterol [16]. Given this, it is not surprising that ApoM protein levels are a good predictor for time to insulin requirement. ApoM also showed a correlation with C-peptide and BMI, although the correlation was negative.

The methods that account for different feature types (GRridge, GRridgesel, multiridge, GRlasso) performed somewhat better than the other methods, although plain lasso also performed well. The various selection methods are very competitive and suggest 50 features usually suffice for the prediction. While models add value to the overall prediction, their use in a clinical setting may be limited given that a base model with only HbA_1c_ already performed moderately well (C=0.71). In part, this can be explained by the aforementioned correlation between clinical markers and biomarkers. For example, adding ApoM on top of HDL-cholesterol will have limited effect on discrimination. Nonetheless, it would be interesting to explore further the identified variables in terms of pathophysiology [6].

Strengths of the current study are the use of multiple omics types in large cohorts and the external validation in one of the cohorts. However, certain limitations need to be addressed. Based on the availability of all three molecular classes, we had relatively small sample sizes in each cohort. This may have limited selection of molecules with more modest effect sizes. The samples in GoDARTS were obtained from participants in a non-fasting state, which may have affected the associations for blood lipids. Although we did not include diabetes duration in our base models, the selection of participants was based on those close to diagnosis of diabetes. We therefore do not expect that this would have affected our results to a significant extent. Finally, we did not see any indication of differences between men and women given that sex was not selected in any of the models except for the unpenalised models.

### Conclusion

In conclusion, by using machine learning approaches we show that insulin requirement risk can be modestly well predicted by predominantly clinical variables. Inclusion of molecular markers improves prognostic performance beyond that of clinical variables by up to 5%. Models based on these variables could be useful for identifying people with diabetes at high risk of rapid progression to treatment intensification in order to target interventions to prevent glycaemic progression and development of complications.

### Supplementary Information

Below is the link to the electronic supplementary material.

Supplementary file1 (PDF 1194 KB)

Supplementary file2 (XLSX 25 KB)

## Data Availability

Summary statistics of lipidomic, proteomic and metabolomic data are available from a Shiny dashboard at https://rhapdata-app.vital-it.ch. The generated metabolomic, lipidomic and proteomic data in the DCS, GoDARTS and All New Diabetics In Scania (ANDIS) are considered sensitive patient data and are therefore not publicly available, in compliance with the European privacy regulations governed by the General Data Protection Regulation (GDPR) and according to limitations included in the informed consents signed by the study participants. For the DCS and GoDARTS proteomics data, restricted access for the proteomics data can be obtained via the European Genome/Phenome archive under accession number EGAD00010002447. Metabolomics and lipidomics (DCS, GoDARTS, ANDIS) data are available upon request by contacting the senior authors (LMtH, lmthart@lumc.nl), ERP (E.Z.Pearson@dundee.ac.uk), JWJB (j.beulens@amsterdamumc.nl) and GAR (g.rutter@imperial.ac.uk). Requests should include name and contact details of the person requesting the data, which molecular data and clinical variables are requested, and the purpose of the request.

## Abbreviations

ApoM: Apolipoprotein M
CCL14/HCC-1: C-C motif chemokine 14
CoRF: Co-data regularised random forest
DCS: Hoorn Diabetes Care System
GoDARTS: Genetics of Diabetes Audit and Research in Tayside Scotland
GRridge: Empirical Bayes group-regularised ridge
GRridgesel: Empirical Bayes group-regularised ridge with selection
GRlasso: Empirical Bayes group-regularised lasso
IL18Ra: IL-18 receptor 1
IndS: Indoxyl-sulfate
Lasso: Least absolute shrinkage and selection operator
MFGM: Milk fat globule-EGF factor 8 protein
NG36: Histone-lysine N-methyltransferase
RET: Proto-oncogene tyrosine-protein kinase receptor
SDMA/ADMA: Asymmetric dimethylarginine
SMAD3: Mothers against decapentaplegic homolog 3
