# Supplementary material for: An omics-based machine learning approach to predict diabetes progression: a RHAPSODY study
Source: Diabetologia. 2024 Feb 19;67(5):885–94. doi: 10.1007/s00125-024-06105-8 (PMC10954972; doi:10.1007/s00125-024-06105-8)
Supplement: Supplementary file 1 — Supplementary file1 (PDF 1194 KB) [file 125_2024_6105_MOESM1_ESM.pdf]

Supplemental figures

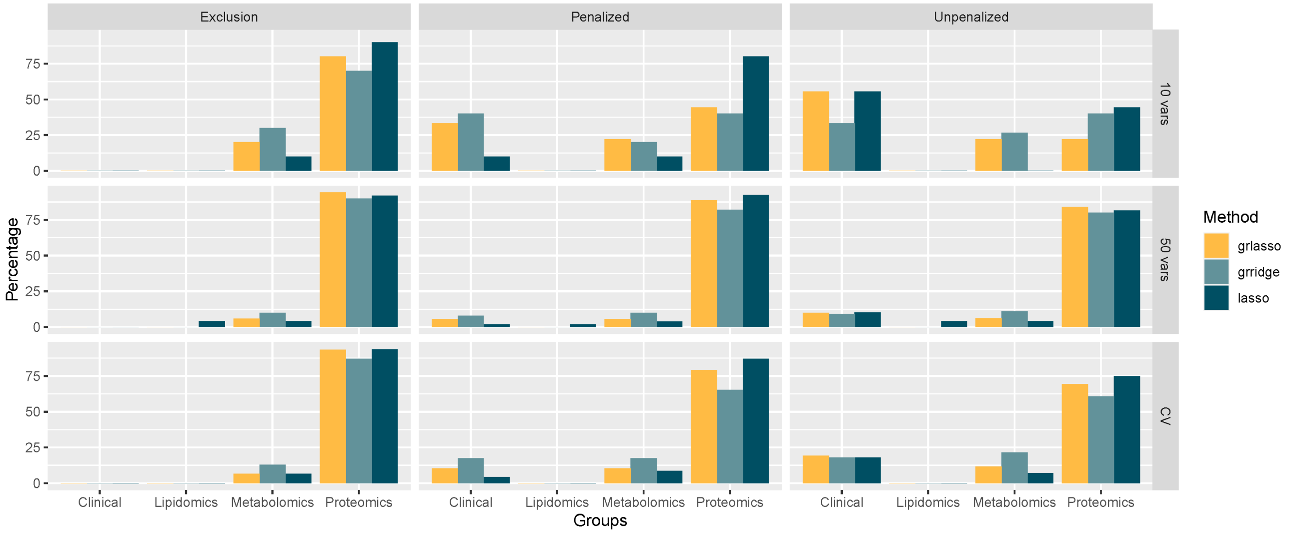

ESM Figure 1. Types of variables selected expressed as percentage of the total number of variables selected. Panels represent the inclusion of clinical variables (exclusion, penalised and unpenalised) and the number of variables selected (10, 50, CV). Colours are the three different methods tested (GRlasso, GRridge, lasso)

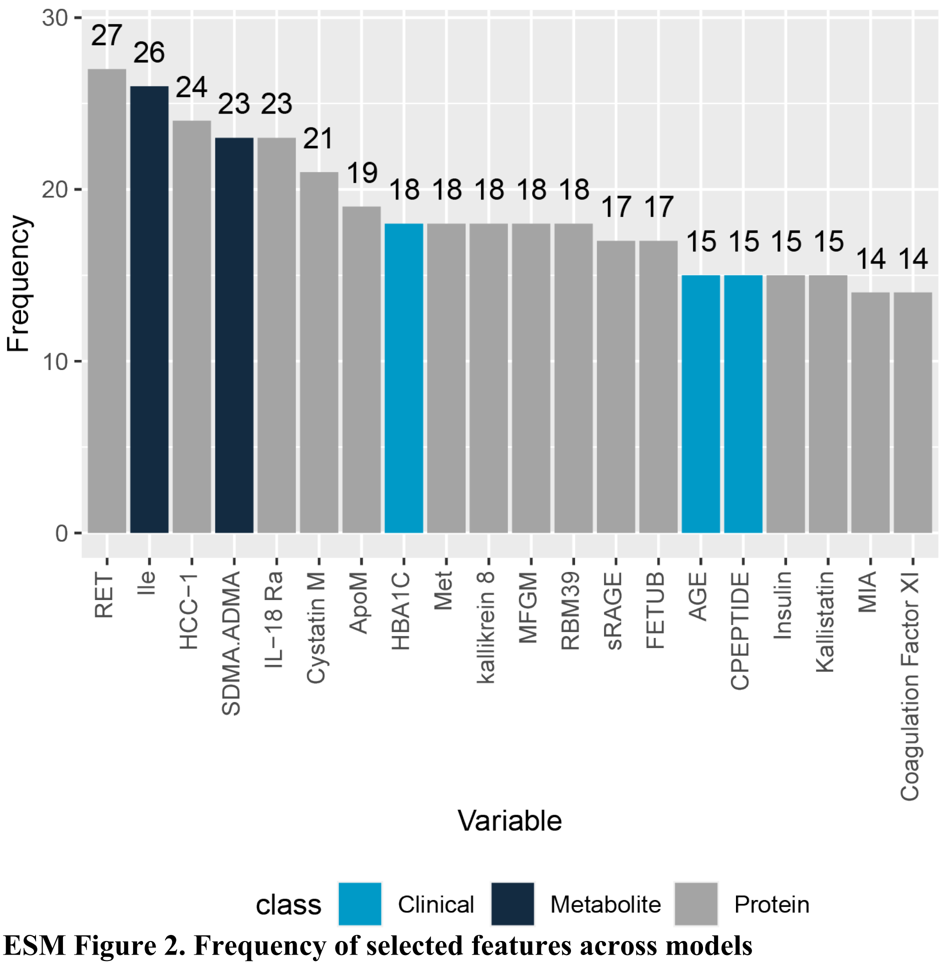

ESM Figure 2. Frequency of selected features across models

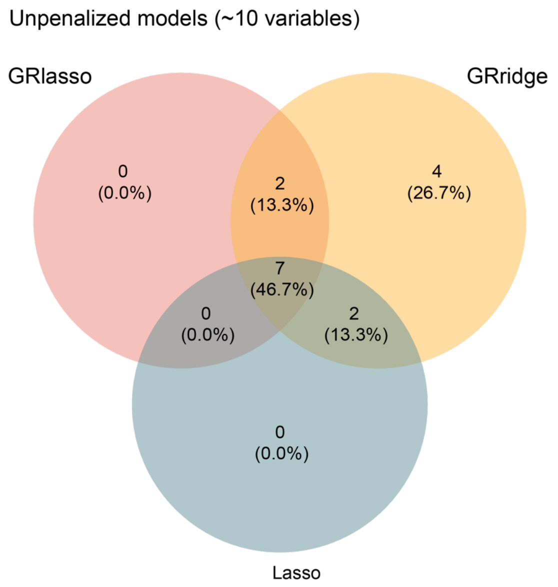

ESM Figure 3. Overlap of the selected variables in the unpenalized models using lasso, GRridge and GRlasso.

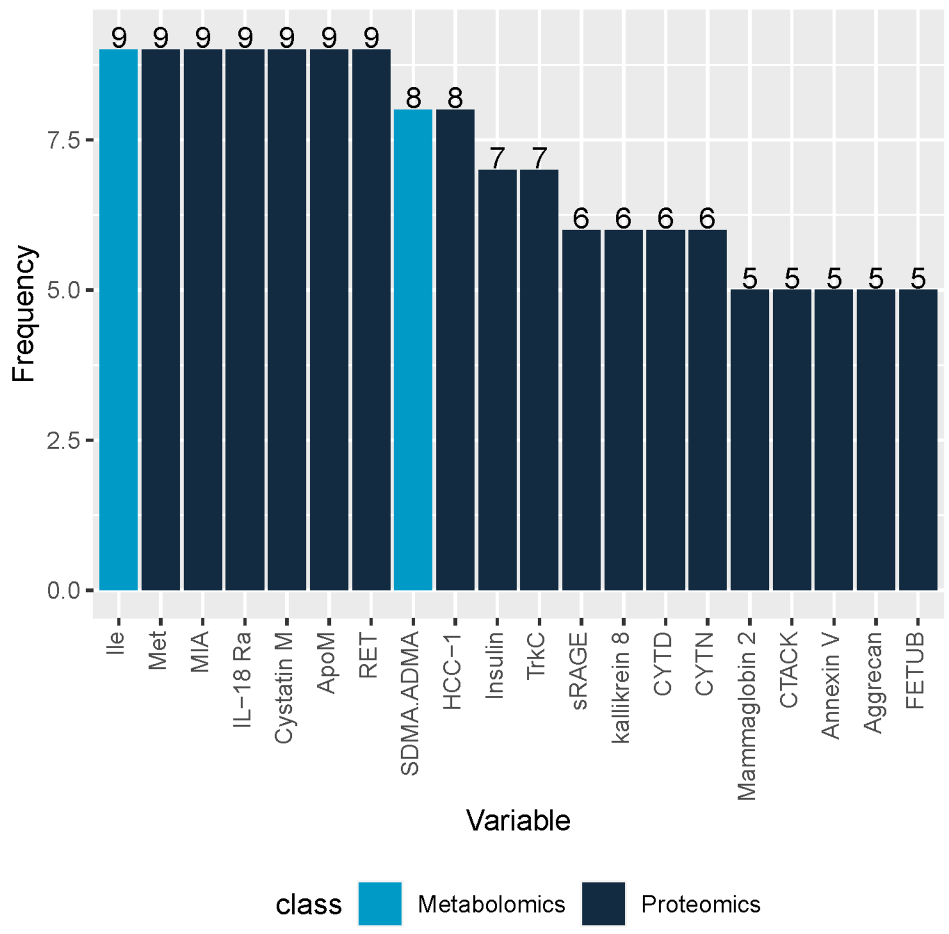

ESM Figure 4. Frequency of variables in the models that excluded the clinical variables.

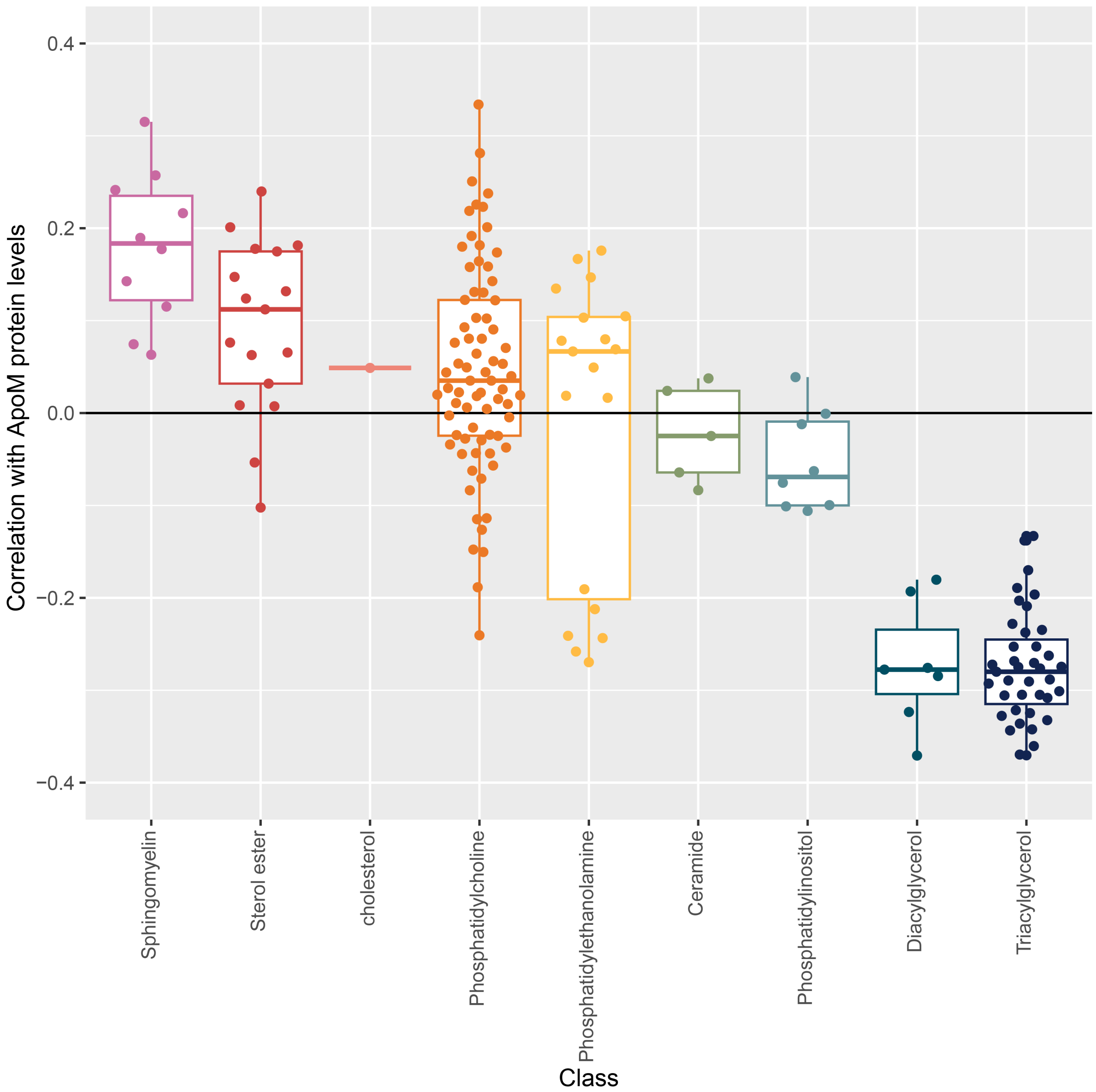

ESM Figure 5. Correlation between plasma ApoM protein levels and plasma lipid levels. X-axis, lipid classes, y-axis correlation with ApoM protein levels and individual lipids. Each dot represents an individual lipid within that class.
